# Chitosan Based Polyelectrolyte Complexes as Potential Carrier Materials in Drug Delivery Systems

**DOI:** 10.3390/md8041305

**Published:** 2010-04-19

**Authors:** Josias H. Hamman

**Affiliations:** Department of Pharmaceutical Sciences, Tshwane University of Technology, Private Bag X680, Pretoria, 0001, South Africa; E-Mail: hammanjh@tut.ac.za; Tel.: +27 12 382 6397; Fax: +27 382 6243

**Keywords:** chitosan, polyelectrolyte complex, self assembly, drug delivery, modified release

## Abstract

Chitosan has been the subject of interest for its use as a polymeric drug carrier material in dosage form design due to its appealing properties such as biocompatibility, biodegradability, low toxicity and relatively low production cost from abundant natural sources. However, one drawback of using this natural polysaccharide in modified release dosage forms for oral administration is its fast dissolution rate in the stomach. Since chitosan is positively charged at low pH values (below its pK_a_ value), it spontaneously associates with negatively charged polyions in solution to form polyelectrolyte complexes. These chitosan based polyelectrolyte complexes exhibit favourable physicochemical properties with preservation of chitosan’s biocompatible characteristics. These complexes are therefore good candidate excipient materials for the design of different types of dosage forms. It is the aim of this review to describe complexation of chitosan with selected natural and synthetic polyanions and to indicate some of the factors that influence the formation and stability of these polyelectrolyte complexes. Furthermore, recent investigations into the use of these complexes as excipients in drug delivery systems such as nano- and microparticles, beads, fibers, sponges and matrix type tablets are briefly described.

## 1. Introduction

Mixing oppositely charged polyelectrolytes in solution will result in their self assembly or spontaneous association due to the formation of strong, but reversible electrostatic links. These direct interactions between the polymeric chains lead to the formation of polyelectrolyte complex networks with non-permanent structures while avoiding the use of covalent cross-linkers. In general, these polymeric networks or hydrogels are well tolerated, biocompatible and are more sensitive to changes in environmental conditions [1,2]. The formation and stability of these polyelectrolyte complexes depend on many factors such as the degree of ionization of each of the oppositely charged polyelectrolytes, the density of the charges on the polyelectrolytes, the charge distribution over the polymeric chains, the concentration of the polyelectrolytes, their mixing ratio, the mixing order, the duration of the interaction, the nature of the ionic groups, the position of the ionic groups on the polymeric chains, the molecular weight of the polyelectrolytes, the polymer chain flexibility as well as the temperature, ionic strength and pH of the reaction medium [3–6]. When polyelectrolytes are mixed in such a ratio that there is an excess of one charge (either positive or negative), a non-stoichiometric complex is formed that are usually soluble. On the other hand, a stoichiometric polyelectrolyte complex contains equal amounts of each opposite charge with a zero net charge in the resultant complex. Stoichiometric polyelectrolyte complexes are usually insoluble and precipitate out of solution upon formation [7].

**Figure 1:**
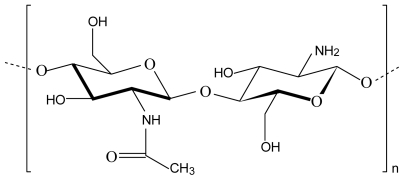
Chemical structure of chitosan consisting of *N*-acetyl-d-glucosamine and glucosamine units.

Chitosan refers to a series of polymers that are deacetylated derivatives of the natural polysaccharide, chitin, with different degrees of deacetylation and molecular weights. It is composed of β-1,4-linked glucosamine (deacetylated units) and *N*-acetyl-d-glusoamine (acetylated units) (Figure 1) with typical degrees of deacetylation between 70 and 95% and molecular weights between 10 and 1,000 kDa [8,9]. Highly refined grades of chitosan have been used in pharmaceutical formulations as a release-controlling agent in oral preparations [10]. Although chitosan is a very promising biopolymer for use as carrier material in drug delivery systems, it has a limited capacity for controlling drug release from oral dosage forms due to its fast dissolution in the stomach. To overcome this disadvantage, chemical modifications such as co-polymerisation or derivatisation have been applied. However, this approach leads to the formation of new chemical entities with unknown toxicological profiles and a physical modification of the polymer is therefore rather preferred. Three dimensional polymeric networks or complexes prepared by non-covalent strategies rely on electrostatic, hydrophobic and/or hydrogen bonding forces between the polymeric chains rather than chemical bonds. These inter-polymer chain interactions are physical in nature and reversible but can provide the required properties for optimal drug delivery if the correct polymers are combined [5,11,12].

The cationic amino groups on the C2 position of the repeating glucopyranose units of chitosan can interact electrostatically with the anionic groups (usually carboxylic acid groups) of other polyions to form polyelectrolyte complexes. Many different polyanions from natural origin (e.g. pectin, alginate, carrageenan, xanthan gum, carboxymethyl cellulose, chondroitin sulphate, dextran sulphate, hyaluronic acid) or synthetic origin (e.g., poly (acrylic acid)), polyphosphoric acid, poly (L-lactide) have been used to form polyelectrolyte complexes with chitosan in order to provide the required physicochemical properties for the design of specific drug delivery systems [2].

Chitosan complexes have been used in a wide range of pharmaceutical applications such as those formed with DNA to serve as non-viral vectors for gene delivery [13] and complexes formed between chitosan and anionic polymers have been investigated for use as biosensors, scaffolds in tissue engineering, for waste-water treatment and for drug delivery in different forms [14]. Most of the investigated polycomplexes that involve chitosan are those formed with other polysaccharides, which are divided into natural polysaccharides (including phytopolysaccharides, zoopolysaccharides and microorganism polysaccharides) and synthetic polysaccharides [15]. This review specifically focuses on polyelectrolyte complexes formed between chitosan and selected natural and synthetic polyanions that have been investigated as excipients in modified release drug delivery systems such as matrix type tablets, beads, sponges, microparticles or nanoparticles for different routes of administration.

## 2. Polyelectrolyte Complexes between Chitosan and Natural Polymers

Many different polyelectrolyte complexes between chitosan and anionic natural polymers have been prepared and investigated. However, some of these polyelectrolyte complexes have been formed and characterised but not yet investigated for drug delivery purposes such as those formed between chitosan and polygalacturonic acid [16], sodium dextransulfate [17], carboxymethyl cashew gum [18], fibroin [19], sodium carboxymethyl cellulose [20] and “angico” gum [21]. Therefore, only the following selected natural polyanions will be described in more detail in terms of their polyelectrolyte complexes with chitosan namely alginate, carrageenan, pectin, xanthan gum, hyaluronic acid, gum kondagogu, gelatine, poly-γ-glutamic acid and maleic starch half-ester. Furthermore, only polyelectrolyte complexes prepared without the addition of chemical cross-linkers are considered here.

### 2.1. Chitosan-alginate polyelectrolyte complex

**Figure 2:**
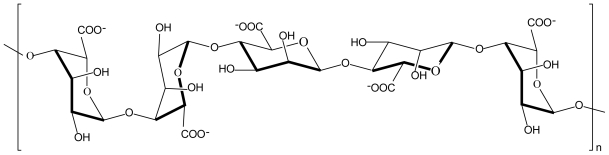
Chemical structure of alginate.

Alginate is a natural, linear, unbranched, biodegradable polysaccharide consisting of 1,4-linked β-d-mannuronic acid and α-l-guluronic acid monomers in varying proportions (Figure 2). Alginates are extracted from brown seaweeds and marine algae such as *Laminaria hyperborea*, *Ascophyllum nodosum* and *Macrocystis pyrifera* [22,23]. The negatively charged carboxylic acid groups of manuronic and guluronic acid units in alginate interact electrostatically with the positively charged amino groups of chitosan to form a polyelectrolyte complex. Alginate is one of the most studied anionic polyelectrolytes in complexation with chitosan because the polyelectrolyte complex formed between these two polymers is still biodegradable and biocompatible but mechanically stronger at lower pH values where chitosan dissolves [24].

A study on the biodegradation of chitosan-alginate polyelectrolyte complexes showed that while chitosan (with a low degree of deacetylation) alone was effectively degraded by lysozymes, the effect of these enzymes on the polyelectrolyte complex was negligible. The polyelectrolyte complex did show a high ability of lysozyme adsorbtion, but enzymatic degradation was hindered by the strong interaction between the chitosan and alginate polymeric chains. However, the polyelectrolyte complexes showed partial degradation by means of hydrolysis. Since the rate of biodegradation may be regulated by changing the polymer ratio, it indicates that this particular polyelectrolyte complex has a high potential in tissue engineering for scaffolds and support materials [25].

It was shown that charge ratio, molecular weight, ionic strength, pH, mixing order as well as speed and diameter of the dispersing element influence the particle size, particle surface charge (zeta potential) and stability of alginate-chitosan polyelectrolyte complexes [26]. In a study wherein chitosan and alginate were reacted in their completely ionised states by maintaining the pH values of each polymer’s solution at a specific value (*i.e.*, pH 2 for the chitosan solution and pH 6.5 for the alginate solution) polyelectrolyte complex beads were formed that showed improved stability of the entrapped α-amylase. The selected pH values ensured an increased charge density on each polymer and led to intense cross-linking during polyelectrolyte complex formation and consequently beads with small micropores were formed. In addition, the chitolytic activity of α-amylases was almost completely suppressed in the acidic environment [22].

Chitosan-alginate polyelectrolyte complex fibers showed promising results for controlling the release of charged molecules and exhibited high encapsulation efficiencies of these molecules [27]. A comparison of matrix type tablets made of the polyelectrolyte complexes formed between chitosan and alginate and those formed between chitosan and carrageenan showed that the former was better in prolonging diltiazem release at lower concentration of the polymers [28].

### 2.2. Chitosan-carrageenan polyelectrolyte complex

**Figure 3:**
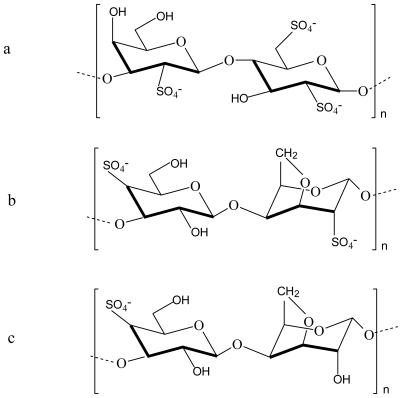
Chemical structures of (a) λ-carrageenan, (b) ι-carrageenan and (c) κ-carrageenan.

Carrageenan is the generic name for a family of high molecular weight sulphated polysaccharides obtained from certain species of red seaweeds. There are three basic types of carrageenan, namely kappa (κ), iota (ι) and lambda (λ) carrageenan (Figure 3) [23,29].

It was shown that the nature or type of carrageenan considerably influence the characteristics of the polyelectrolyte complex that is formed with chitosan. The mechanical strength of polyelectrolyte complex gels formed between chitosan and different carrageenans were in the order λ- > ι- > κ-carrageenan and although the latter two formed stronger gels due to the formation of more cross-links as a result of their double helix secondary structures, these gels were also more brittle. Furthermore, the gels obtained for ι- and κ-carrageenan were temperature sensitive because of the helix coil conformational transitions in their molecules [30].

An investigation was done on the potential of polyelectrolyte complexes between κ-, ι-, λ-carrageenan and chitosan to form controlled release systems for glucose oxidase. The complex between chitosan and κ-carrageenan showed high encapsulation efficiencies for glucose oxidase while having the lowest release rate for this compound. Furthermore, this complex was able to protect the encapsulated glucose oxidase against degradation in pH 1.2 solution, in a chitosanase solution and in a pepsin solution [31].

### 2.3. Chitosan-pectin polyelectrolyte complex

**Figure 4:**
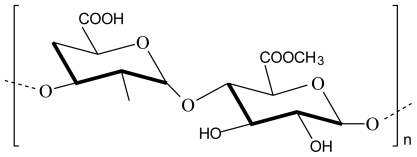
Chemical structure of pectin.

The polysaccharides of the plant cell wall consist mainly of cellulose, hemicelluloses and pectin. Pectin is a linear polysaccharide composed of α-1,4-linked D-galacturonic acid units, however, this linear structure is interrupted with highly branched regions in the polymer chain (Figure 4). The composition of the pectin molecule varies from source to source, e.g. pectin from citrus fruit contains less neutral sugars and has a smaller molecular size than pectin from apples [32–34].

When acidic chitosan and pectin solutions are mixed a homogenous solution is obtained without any ionic interactions between the two polymers. A polyelectrolyte complex could be obtained by adjusting the pH of this mixture to a value of 5.5 where electrostatic interactions between the negatively charged carboxylic acid groups of pectin and the positively charged amino groups of chitosan occurred. As expected, the extent of this interaction depended on the pH of the surrounding medium which determined the extent of ionization of the polymers [14].

Polyelectrolyte complexes composed of chitosan and pectin were compressed into tablets with vancomycin as model drug. The tablets showed a pH sensitive swelling ability with drug release behaviour based on enzyme dependent degradation in the presence of beta-glucosidase, which indicated potential for colon-specific delivery of vancomycin [35].

### 2.4. Chitosan-xanthan gum polyelectrolyte complex

**Figure 5:**
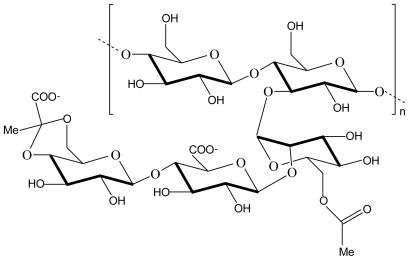
Chemical structure of xanthan gum.

Xanthan gum is an exopolysaccharide secreted from *Xanthomonas campestris*. It consists of a cellulosic backbone, namely β-(1,4)-d-glucopyranose glucan, with a trisaccharide side chain, namely (3,1)-α-d-mannopyranose-(2,1)-β-d-glucuronic acid-(4,1)-β-d-mannopyranose, on every second glucose residue (Figure 5) [36].

Results obtained from a modulated differential scanning calorimetry analysis and the swelling degree of microcapsules prepared from chitosan-xanthan gum polyelectrolyte complexes indicated that the cross-linking density was interdependent on xanthan concentration, chitosan concentration and chitosan solution pH [37]. A kinetic analysis through rheological measurements of chitosan-xanthan polyelectrolyte complex formation showed that the coacervate is formed in two distinctive steps. Furthermore, the kinetic curve showed a classic sol-gel transition and the hydrogel is organised in a quasi-ordered network during coacervation. After a period of mixing the hydrogel showed a structural modification and a stable storage modulus with solid-like behaviour was obtained. Mechanical grinding of the freeze-dried polyelectrolyte complex resulted in a powder with particles that have a suitable diameter for pharmaceutical applications [38].

Chitosan-xanthan polyelectrolyte complexes have been investigated as a matrix for enzyme immobilisation [39,40], but a unique application of this matrix in the pharmaceutical field is to enhance the dissolution of water insoluble drugs such as fenofibrate, ursodeoxycholic acid, nifedipine and indomethacin [38].

### 2.5. Chitosan-hyaluronic acid polyelectrolyte complex

**Figure 6:**
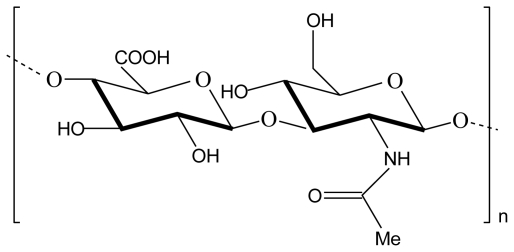
Chemical structure of hyaluronic acid.

Hyaluronic acid (also called hyaluronan or hyaluronate) is the only nonsulfated glycosaminoglycan found in the extracellular matrix throughout connective, epithelial and neural tissues. Hyaluronic acid is a linear anionic polysaccharide with high molecular weight that consists of β (1,3)-*N*-acetyl-d-glucosamine and α (1,4)-d-glucuronic acid repeating units linked by β (1→3) bonds (Figure 6). It is produced through bacterial fermentation of streptococcus species or extracted from rooster combs, umbilical cords, synovial fluids or vitreous humour for commercial purposes. It has been used in ophthalmic surgery, arthritis treatment, in tissue engineering, a component of scaffolds for wound healing and implant devices [8,41,42].

It was shown that the polyelectrolyte complex between chitosan and hyaluronic acid protected hyaluronic acid against enzymatic hydrolysis, but only at pH values different from the optimal pH of the enzyme. The results from this study revealed that the chitosan-hyaluronic acid polyelectrolyte complex unfortunately had less cell proliferation and wound healing effects compared to chitosan alone [43].

Chitosan-hyaluronic acid polyelectrolyte complex beads were prepared and these beads were reacted in another step with hyaluronic acid to form an outer layer around the bead, which increased their stability [44]. Furthermore, when hyaluronic acid was incorporated into the hydrogel prepared from chitosan and poly (*N*-isopropylacrylamide) it prevented disintegration of the system during *in vitro* drug release studies and thereby facilitated controlled release of nalbuphine [45].

Chitosan-hyaluronic acid polyelectrolyte complex nanoparticles loaded with heparin were effectively internalised by rat mast cells and showed similar activity in terms of preventing histamine release compared to free heparin. A high degree of loading efficiency was observed (approximately 70%) and the nanoparticles were stable in phosphate buffered saline for at least 24 h. The release of unfractionated heparin was much slower (10.8% within 12 h) than that of low molecular weight heparin (79.7% within 12 h). These results indicated the usefulness of these nanoparticles to serve as effective delivery systems for pulmonary administration [46].

### 2.6. Chitosan-gum kondagogu polyelectrolyte complex

Gum kondagogu is a natural biopolymer that is obtained from the exudated gum of *Cochlospermum gossypium*, a tree growing naturally in India. It consists of a complex polysaccharide with a relatively high molecular weight (ranging between 9.8 × 10^5^ and 2.5 × 10^7^ g/mol) and therefore exhibit a high viscosity in solution with gelation characteristics. When the acid hydrolysed gum was characterised by gas chromatography linked to mass spectrometry (GC-MS), the following monosaccharides were indicated to be present: rhamnose, galacturonic acid, glucuronic acid, β-d-galactopyranose, α-d-glucose, galactose, arabinose, mannose and fructose [47,48].

Chitosan-gum kondagogu polyelectrolyte complex microparticles loaded with diclofenac sodium showed controlled drug release, which was depended on the pH of the dissolution medium. A pharmacokinetic study of these microparticles in rats revealed that although the time to reach the peak plasma concentration (T_max_) was higher compared to that of free dicolfenac sodium, the extent of drug absorption (area under the concentration-time curve) was also increased significantly when administered in the form of the microparticles [11].

### 2.7. Chitosan-gelatine polyelectrolyte complex

**Figure 7:**
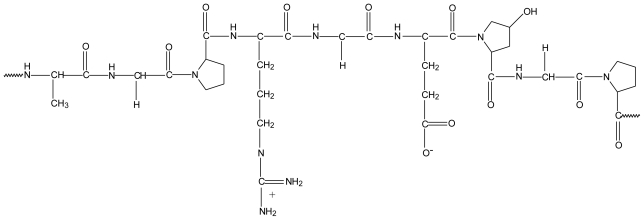
Typical structure of gelatin.

Gelatine is a heterogeneous mixture of protein fractions consisting of single or multi-stranded polypeptides (Figure 7). It is obtained by partial hydrolysis of animal collagen derived from skin, white connective tissues and bones. Type A gelatine is derived from pig skin by means of acid hydrolysis and type B gelatine from alkaline hydrolysis of cattle hides and bones [49].

It was shown that the polyelectrolyte complex between chitosan and gelatine can only occur at a pH value above 4.7 (which represents the isoelectric point of gelatin and above this value the net charge on gelatine type B is negative) and below 6.2 (above which chitosan start to precipitate out of solution) [50]. Chitosan-gelatine polyelectrolyte complex sponges containing tramadol hydrochloride were found to retard drug release, which followed Higuchi’s diffusion mechanism over a 12 h period. Subcutaneous implants of these formulations in rats showed reasonable analgesic effect that could be maintained for more than 8 h. Furthermore, the polyelectrolyte complex between chitosan and gelatine produced sponges with improved mechanical properties compared to sponges containing chitosan alone [51].

### 2.8. Chitosan-γ-poly(glutamic acid) polyelectrolyte complex

Gamma poly (glutamic acid) is a hydrophilic polyanion that is water-soluble, non-toxic and biodegradable. This polyamino acid is formed by amide bond linkages between the amino group on theα carbon atom of one glutamic acid molecule and the carboxyl group on the γ carbon atom of the next glutamic acid molecule [52].

It was shown that stable nano-sized particles can be formed as a direct result of interactions between the carboxylic groups of linear γ-poly (glutamic acid) chains and the amino groups of linear chitosan. The size and stability of the nanoparticles depended on factors such as medium pH, concentration and ratio of γ-poly (glutamic acid) and chitosan solutions as well as the mixing order [53].

In a study wherein chitosan-γ-poly (glutamic acid) polyelectrolyte complex nanoparticles were investigated as drug carriers for targeted drug delivery, it was shown that they can penetrate A2780/AD ovarian cancer cells and this penetration was significantly faster and more efficient in the presence of conjugated folic acid due to over expression of folate receptors on these cells [54]. It was shown that insulin loaded chitosan-γ-poly (glutamic acid) polyelectrolyte complex nanoparticles caused a significant hypoglycemic action in diabetic rats with a relative oral insulin bioavailability of 15.1% [55]. In another study insulin containing chitosan-γ-poly (glutamic acid) polyelectrolyte complex nanoparticles were filled into enteric coated capsules that resulted in approximately 20% relative bioavailability [56]. However, it should be mentioned that in these *in vivo* studies that investigated delivery of encapsulated insulin, the nanoparticles were formed by self-assembly of chitosan and γ-poly (glutamic acid) in the presence of the cross-linkers such as tripolyphosphate and magnesium sulphate.

### 2.9. Chitosan-maleic starch half-ester acid polyelectrolyte complex

Maleic starch half-ester acid is an anionic derivative of starch that produces a polyelectrolyte complex when combined with the polycation, chitosan. Characterisation and evaluation of this complex indicated the driving force for self-assembly was electrostatic interactions. A pH sensitive release of 5-fluorouracil was obtained that was also dependent on the maleic starch half-ester to chitosan ratio as well as the carboxyl content of maleic starch half-ester used as starting material [57].

## 3. Polyelectrolyte Complexes between Chitosan and Synthetic Polymers

### 3.1. Chitosan-cross-linked-poly(acrylic acid) polyelectrolyte complexes

Polycarbophil (Noveon AA-1^®^) and Carbopol are high molecular weight polymers consisting of acrylic acid monomers. The poly (acrylic acid) chains are cross-linked with divinyl glycol to form polycarbophil and polyalkenyl alcohols such as allyl ethers of pentaerythritol or allyl ethers of sucrose to form Carbopol. Cross-linking of the poly (acrylic acid) chains with divinyl glycol in the case of polycarbophil renders only 20% of the carboxylic acid groups inaccessible for interactions with other compounds [58].

The main mechanism of complexation between chitosan and Carbopol was found to be an electrostatic interaction between the NH_3_^+^ groups of chitosan and the COO^−^ groups of Carbopol as determined by FT-IR analysis. The ratio of polymers involved in polyelectrolyte complex formation could be controlled by the pH of the medium. Drug release from matrix type tablets prepared from polyelectrolyte complexes consisting of different chitosan to Carbopol ratios did not show significant differences at pH 1.2, but differences were clearly shown at pH 6.8 [59]. A matrix type tablet prepared from the polyelectrolyte complex between chitosan and Carbopol containing theophylline as model drug exhibited a similar drug release profile as matrix type tablets prepared from hydroxypropyl methyl cellulose. The drug release was pH dependent with a diffusional release mechanism at pH 6.8 and a relaxational release mechanism at pH 1.2 [60].

Films for dermal drug delivery were prepared from the polyelectrolyte complexes between chitosan and polycarbophil (Noveon AA-1^®^) as well as between chitosan and Carbopol 71G NF^®^, which showed promising characteristics in terms of flexibility, resistance and water vapour transmission rate. These films showed such great potential as formulations for topical and transdermal drug administration during this characterization study that it was suggested they should be investigated for incorporation, release and delivery of drugs [61].

Monolithic matrix type tablets prepared from chitosan-polycarbophil (Noveon AA-1^®^) polyelectrolyte complex as matrix former approached zero order release for two model drugs with different water solubilities (*i.e.*, diltiazem and ibuprofen). The rate of the release of these two model drugs depended on the concentration of the polyelectrolyte complex in the formulation of the matrix systems. When the matrix type tablets consisted only of polyelectrolyte complex (without drug), they showed a very high swelling capability without erosion, which means the swelling is completely reversible upon drying without any weight loss [62]. In another study it was shown that matrix type tablets prepared from the polyelectrolyte complex between chitosan and polycarbophil (Noveon AA-1^®^) were capable of slower drug release than those prepared from hydroxypropylmethylcellulose [63]. When insulin was included as model drug in matrix systems containing the chitosan-polycarbophil (Noveon AA-1^®^) polyelectrolyte complex, it was shown that the mechanism of release was dependent on the concentration of the polyelectrolyte complex in the formulation of the matrix systems relative to the other excipients. The matrix systems which contained the highest concentration of the polyelectrolyte complex approached zero-order insulin release and exhibited erosion in conjunction with swelling [64]. In general, the polyelectrolyte complex between chitosan and polycarbophil has shown great potential as a matrix former for modified release dosage forms according to *in vitro* drug release results.

### 3.2. Chitosan-polymethacrylate copolymer (Eudragit) polyelectrolyte complexes

Polymethacrylate copolymers (or Eudragit) are well known film-coating agents for oral dosage forms such as capsules or tablets. Different types of Eudragit polymers exist which are synthetic methacrylic acid copolymers consisting of different ratios of dimethylaminoethylmethacrylates, methacrylic acid and methacrylic acid esters. Some of them are polycations (Eudragit E, RL, RS and NE) due to the presence of dimethylamino groups or quaternary amino groups, while others are polyanions (Eudragit L and S) due to the presence of carboxylate groups [65,66].

The first reference to an ionic interaction between chitosan and Eudragit was in a study where chitosan microcores containing sodium diclofenac as model drug were microencapsulated in Eudragit S-100 using an oil-in-oil solvent evaporation technique for colon targeted release. The ionic interaction between the two polymers was confirmed by means of infrared spectroscopy and had an influence on the drug release profile from the microspheres [67].

Polyelectrolyte complexes formed between chitosan with different molecular weights and Eudragit L100 or Eudragit L100-55 were compressed into matrix type tablets containing diclofenac sodium as model drug. Different aspects of the complex systems were investigated such as the molar ratio, structure, swelling and drug release profiles. Results indicated that this polyelectrolyte complex has high potential for manufacture of controlled release drug delivery systems. Factors such as the composition of the polyelectrolyte complex in terms of polymer ratios, Eudragit type and molecular weight of the chitosan influenced the drug release rate from the matrix type tablets [12].

### 3.3. Chitosan-polyalkylenoxide-maleic acid polyelectrolyte complex

**Figure 11:**
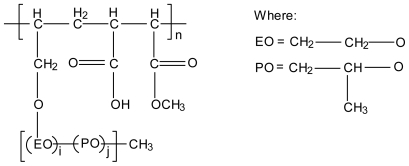
Chemical structure of polyalkylenoxide-maleic acid copolymer.

By changing the alkyleneoxide chain composition, a series of polyelkylenoxide-maleic acid (PAOMA) copolymers can be synthesised with different characteristics (e.g., hydrophilic or hydrophobic, Figure 11). PAOMA is an anionic polymer that was used to form polyelectrolyte complexes with chitosan in the form of films containing salicylic acid and phenol as model drugs and their drug release rates were evaluated. These films indicated pH dependent drug release behaviour and the hydrophobic PAOMA polymer also showed temperature sensitive drug release behaviour [68].

The ethylene oxide (EO) ratio determines whether the copolymer is hydrophilic or hydrophobic. EO ratio means the molar fraction of ethylene oxide in alkylene oxide chain defined by *i*/(*i*+*j*) where *i* and *j* represent the random copolymerisation degree.

## 4. Conclusions

The scope of polymers used in dosage form design can be increased by several approaches such as modification of their chemical structure, by combining different polymers in physical mixtures or by formation of polymer-polymer associations such as polyelectrolyte complexes. Polyelectrolyte complexes combine unique physicochemical properties of different polymers with the advantage of retaining high biocompatibility. It is therefore not surprising that polyelectrolyte complexes are gaining importance in modern pharmaceutical technology [69].

From the *in vitro* studies conducted on chitosan-based polyelectrolyte complexes it is clear that they are valuable excipients with specific properties for efficient dosage form design, which may be valuable in the development of modified drug delivery systems. Unfortunately, the literature lacks *in vivo* data in terms of drug delivery from these dosage forms which makes it difficult to be conclusive in terms of their effectiveness as drug carriers at this stage. Since some work has been done on *in vitro-in vivo* correlations with chemically cross-linked chitosan hydrogels with successful sustained drug delivery in animals (e.g., [70]), it is anticipated that optimized chitosan based polyelectrolyte complexes may also perform up to expectation for *in vivo* drug delivery.

## Figures and Tables

**Figure 8:**
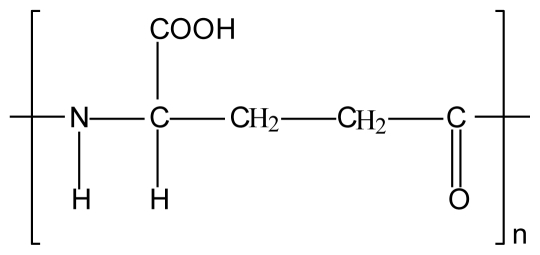
Chemical structure of γ-poly (glutamic acid).

**Figure 9:**
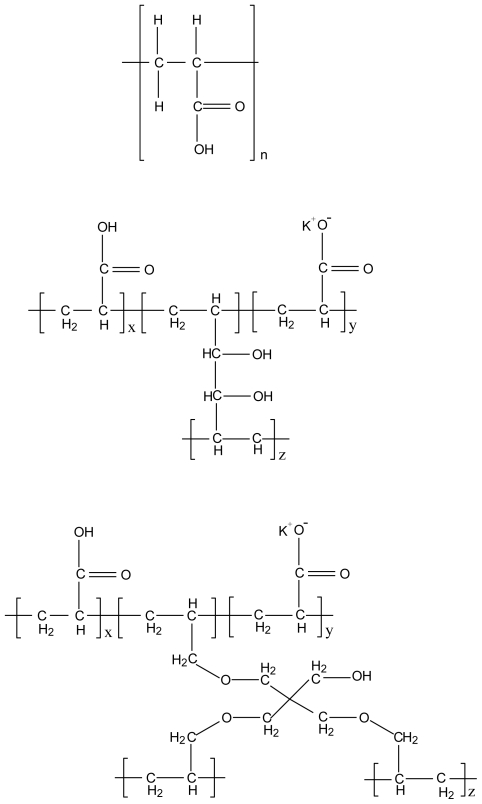
Chemical structure of (a) poly (acrylic acid), (b) polycarbophil AA-1 and (c) Carbopol 974 NF.

**Figure 10:**
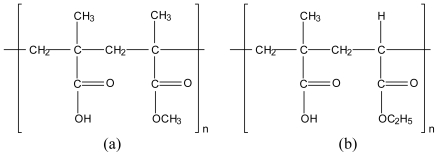
Chemical structure of (a) Eudragit L100 and (b) L100-55.
